# Long-term outcomes of HBsAg/anti-HBs double-positive versus HBsAg single-positive patients with chronic hepatitis B

**DOI:** 10.1038/s41598-019-56015-8

**Published:** 2019-12-19

**Authors:** Min-Sun Kwak, Goh-Eun Chung, Jong In Yang, Jeong Yoon Yim

**Affiliations:** Department of Internal Medicine, Healthcare Research Institute, Healthcare System Gangnam Center, Seoul National University Hospital, Seoul, Republic of Korea

**Keywords:** Hepatitis B, Viral infection, Hepatitis B

## Abstract

The coexistence of HBsAg and anti-HBs has been reported in some chronic hepatitis B patients; however, the long-term outcomes of this serological profile have not been elucidated. We aimed to evaluate the long-term outcomes of HBsAg/anti-HBs double-positive chronic hepatitis B patients. Chronic hepatitis B patients who underwent baseline abdominal ultrasonography and follow-up (HBsAg/anti-HBs assessment and abdominal ultrasonography) at our healthcare center were included. The “coexistence group” included patients positive for both HBsAg and anti-HBs and the “control group” included patients positive for only HBsAg during follow-up. The outcomes were hepatocellular carcinoma (HCC) incidence, HBsAg seroclearance and overall mortality. Kaplan-Meier and Cox proportional hazard regression analyses were performed. Of the 2,341 eligible patients, 166 (7.1%) were in the coexistence group. The total follow-up duration was 5.4 years. The coexistence group had a 3.08-fold higher risk of HCC than the control group [hazard ratio (HR) 3.08, 95% confidence interval(CI) 1.26–7.55, P = 0.014] in multivariate analysis. The coexistence group had more HBsAg seroclearance than the control group (HR 1.43, 95% CI 1.01–2.03, P = 0.046). However, overall mortality did not significantly differ between the 2 groups. The coexistence group is heterogeneous and includes subjects with unfavorable outcomes (incidence of HCC) and favorable outcomes (more HBsAg seroclearance).

## Introduction

The natural course of hepatitis B virus (HBV) infection is determined by the interrelationship between viral replication and host immunity. Thus, the serological detection of viral proteins (hepatitis B surface antigen (HBsAg)) and host-produced antibodies (anti-HBs) is usually performed to evaluate the status of HBV infection. Typically, the emergence of anti-HBs indicates infection recovery as antibodies against HBsAg can neutralize HBV, which clears up circulating HBsAg and infectious HBV particles from peripheral blood^1^. However, the HBsAg/anti-HBs double-positive serological profile is observed in several clinical situations.

Recently, the prevalence of HBsAg and anti-HBs double-positive serological profiles was reported to vary from 2.8 to 3.6% among different cohorts^2–5^. In Korea, a large-scale survey from multiple health check-up institutions showed a 2.9% coexistence of anti-HBs in the HBsAg-positive group^2^. However, the clinical significance and long-term outcomes of this coexistence of HBsAg and anti-HBs has not been well established. A study by Jang *et al*. showed that concurrent HBsAg and anti-HBs are risk factors for the development of hepatocellular carcinoma (HCC)^6^. Heijtink *et al*. suggested that this serological profile is histologically associated with advanced chronic liver disease^7^. In contrast, several other studies have showed no association between the coexistence of HBsAg and anti-HBs and the severity of chronic liver disease^8,9^. Therefore, the aim of this study was to determine the long-term outcomes of the coexistence of HBsAg and anti-HBs.

## Patients and Methods

### Patients

We included subjects with chronic HBV infection, defined by HBsAg positivity for >6 months, who underwent abdominal ultrasonography at baseline and repeated measurements including HBsAg, anti-HBs, alpha-fetoprotein (AFP) and abdominal ultrasonography every 6 months-2 years at Seoul National University Hospital Healthcare System Gangnam Center between 2003 and 2018. We excluded patients coinfected with hepatitis C virus or human immunodeficiency virus (determined by the presence of anti-hepatitis C antibodies or anti- human immunodeficiency virus antibodies). We also excluded patients with HCC or other malignancies at baseline. Patients with a history of liver transplantation were also excluded.

This study protocol conformed to the ethical guidelines of the 1975 Declaration of Helsinki and was approved by the Institutional Review Board of Seoul National University Hospital (H-1704-078-846). The requirement for informed consent from subjects was waived by the Institutional Review Board of Seoul National University Hospital because the researchers accessed only deidentified database entries for analytical purposes.

### Clinical and laboratory assessments

Demographic data, medication history including antiviral agents and comorbidities such as malignancy were assessed based on electronic medical records.

Laboratory tests included assessments of serology associated with HBV infection, liver function, AFP levels, platelet counts, and antibodies against hepatitis C virus and human immunodeficiency virus. Serologic markers for HBV including HBsAg, anti-HBs, hepatitis B e antigen (HBeAg), and anti-HBe were assessed by chemiluminescent microparticle immunoassays (Abbott Architect i2000SR System, IL, USA). The concentration of HBsAg was determined using a previously generated Architect HBsAg calibration curve (range, 0.05–250 IU/ml), and the samples with higher than 250 IU/ml HBsAg levels were diluted to 1:500–1:1000. By June 2010, qHBsAg more than 250 were expressed as >250 IU/ml without presenting an exact value. Thus, we divided subjects into 2 groups as those with qHBsAg > 250 IU/ml and those with qHBsAg ≤ 250 IU/ml in this study.

Serum HBV DNA levels were measured with Roche COBAS TaqMan (Roche Molecular System, Branchburg, NJ, USA) quantitative PCR assay, which has a low detection limit of 20 IU/mL. The threshold for anti-HBs positivity was an anti-HBs titer >10 IU/mL. Blood samples were collected before 10:00 AM after the patients had completed a 12-h overnight fast. All laboratory tests were conducted using standard methods.

### Definition of HCC occurrence, cirrhosis and significant fibrosis

HCC was diagnosed according to the guidelines of the American Association for the Study of Liver Disease^10^. The presence of cirrhosis was reviewed according to imaging findings of cirrhotic hepatic change and/or clinical criteria indicating portal hypertension, such as the presence of ascites, esophageal or gastric varix, or splenomegaly with thrombocytopenia.

Hepatic fibrosis was defined using a noninvasive prediction model Fibrosis-4 (FIB-4) index. The equation for FIB-4 index is as follows;

FIB-4 = age (years) x AST (U/L)/(platelets (10^9^/L) x [ALT (U/L)] ^1/2^)

Subjects with FIB-4 > 1.45 were regarded as those who have high probability of advanced fibrosis^11,12^.

### Definition of groups and outcome measures

The coexistence of HBsAg and anti-HBs was defined by the simultaneous positivity for both HBsAg and anti-HBs. The “coexistence group” included chronic hepatitis B patients who were positive for both HBsAg and anti-HBs during follow-up. The transient or intermittent coexistence of HBsAg and anti-HBs during follow-up was also included in the “coexistence group”. The dynamic change of HBsAg and anti-HBs of the patients in coexistence group is presented in Supplementary Fig. 1. The “control group” include patients who never experienced coexistence of HBsAg and anti-HBs (HBsAg single-positive) during follow-up.

The primary outcome was HCC incidence. Secondary outcomes were HBsAg seroclearance and all-cause mortality. HBsAg seroclearance was defined as the loss of serum HBsAg, not related to liver transplantation. The follow-up duration for HCC occurrence was calculated from the time of initial visit to the time of last evaluation of the image or the diagnosis of HCC. The follow-up duration for HBsAg seroclearance was calculated from the time of initial evaluation of HBsAg to the time of last HBsAg evaluation or HBsAg seroclearance. Predictors for outcomes were evaluated. All-cause mortality was evaluated in all subjects using the survival data provided by the National Micro Data Service System (Seoul, Korea).

### Statistical analyses

Continuous variables were expressed as the means ± standard deviations, and categorical variables were expressed as numbers and percentages. Between-group comparisons were performed using Student’s *t*-tests or Mann-Whitney U tests for continuous variables and Chi-square tests or Fisher’s exact tests for categorical variables.

Kaplan-Meier graphs were drawn, and log-rank tests were performed to compare the time to HCC incidence, HBsAg seroclearance and death due to any cause. Cox proportional hazard regression analysis was performed to identify the predictors of outcomes. As a sensitivity analysis, we also performed an analysis with propensity score (PS) matching to investigate if the coexistence with HBsAg/Ab affect the incidence of HCC. PS was generated by using a logistic regression analysis to compute the probabilities of HBsAg/Ab coexistence, as a function of 21 covariates which are listed in Supplementary Table 1. Those in the coexistence group were matched (1:1) individually with those in control group based on PS. All the included variables were balanced between 2 groups. All statistical analyses were performed using Stata 14.2 (StataCorp, College Station, TX, USA) and SPSS 21 (SPSS Inc., Chicago, IL, USA), and P-values < 0.05 were considered statistically significant.

## Results

### Data at baseline and during follow-up

**Figure 1:**
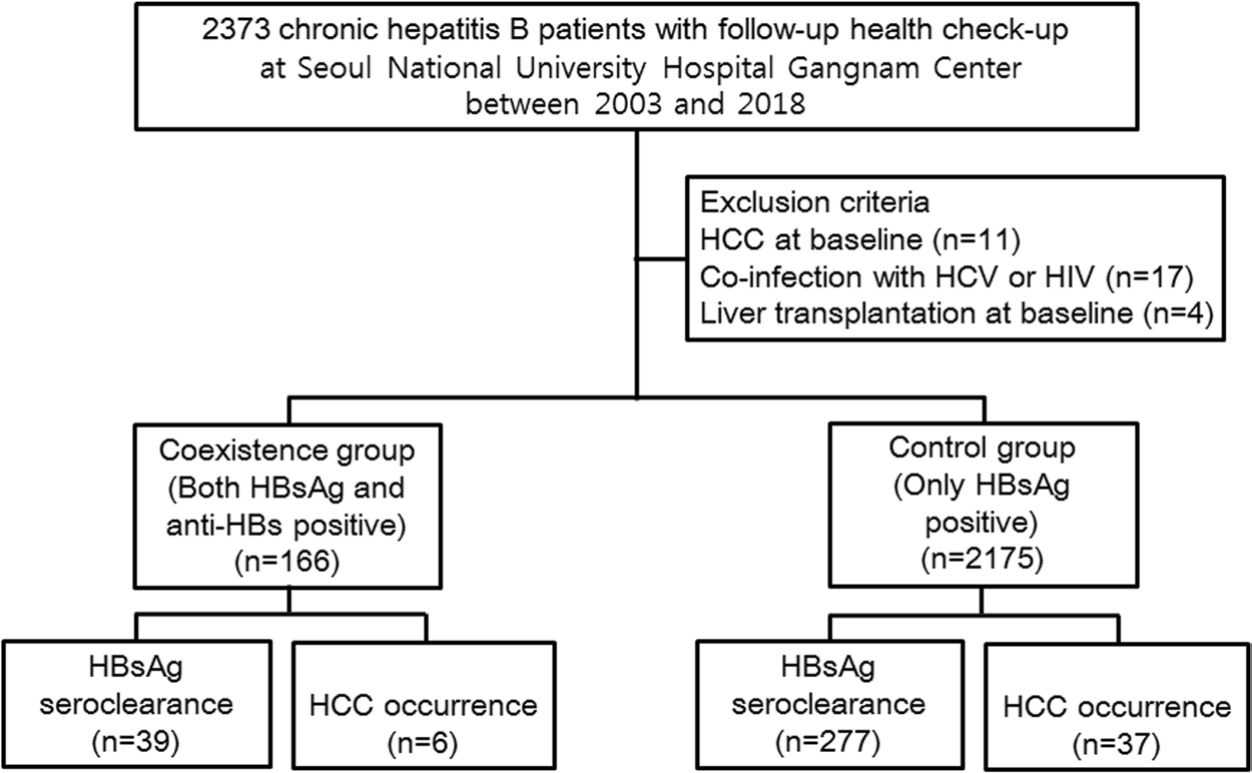
Flowchart of study enrollment.

In total, 2,341 subjects were included in this study. Figure 1 shows a flowchart of study enrollment. The median age of the patients was 46.4 years, and 1,412 subjects were male (60.3%). The coexistence group accounted for 7.1% of the total population. Table 1 shows the baseline characteristics of the overall cohort, coexistence group and control group. There were no significant differences in age, sex, alanine aminotransferase (ALT) level, aspartate aminotransferase (AST) level, presence of cirrhosis, FIB-4 or history of antiviral medication. The follow-up durations were not different between the groups. Less subjects with qHBsAg > 250 IU/ml were included in the coexistence group.

**Table 1:** Baseline characteristics of subjects with coexistence of HBsAg and anti-HBs compared to subjects with positive HBsAg.

	Total (n = 2,341)	Control group (n = 2,175)	Coexistence group (n = 166)	*P*-value
Age, years	46.4 ± 9.3	46.4 ± 9.2	46.7 ± 10.0	0.681
Male, *n* (%)	1412 (60.3%)	1312 (60.3%)	100 (60.2%)	1.000
AST, IU/L	30.4 ± 31.0	30.3 ± 31.1	32.6 ± 30.1	0.345
ALT, IU/L	35.3 ± 50.1	35.1 ± 50.4	38.2 ± 44.8	0.432
qHBsAg > 250 IU/ml, *n* (%)	1930 (82.4%)	1827 (84.0%)	103 (62.0%)	<0.001
FIB-4 > 1.45, *n* (%)	623/2324 (26.6%)	579/2161 (26.8%)	44/163 (27.0%)	0.956
Presence of liver cirrhosis, *n* (%)	76/2334 (3.3%)	74/2170 (3.4%)	2/164 (1.2%)	0.169
Treatment with antiviral agent, *n* (%)	231/2333 (9.9%)	218/2174 (10.0%)	13/159 (8.2%)	0.581
Follow-up duration until last HBsAg evaluation, months	64.7 ± 43.8	64.7 ± 43.7	64.2 ± 45.2	0.883
Follow-up duration until HBsAg seroclearance, months	59.6 ± 41.1	59.8 ± 41.0	57.3 ± 42.4	0.456
Follow-up duration until HCC occurrence, months	58.3 ± 40.4	58.3 ± 40.2	57.4 ± 42.6	0.786
Follow-up duration until mortality, months	124.1 ± 37.7	124.1 ± 37.5	123.4 ± 40.5	0.799

AST, aspartate aminotransferase; ALT, alanine aminotransferase; qHBsAg, quantitative HBsAg; FIB-4, fibrosis-4; HCC, hepatocellular carcinoma.

### Incidence of HCC

**Figure 2:**
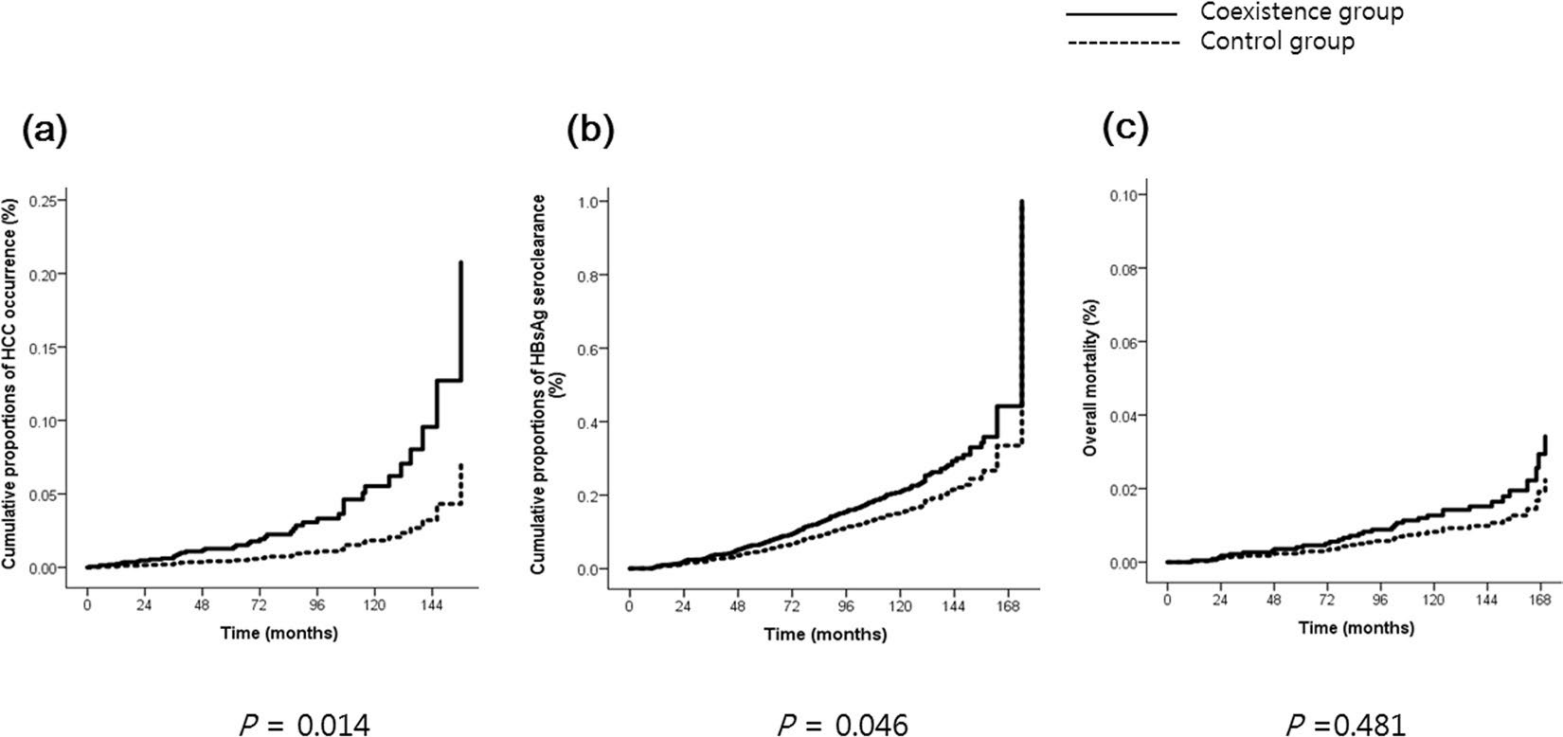
Long-term outcomes including (**a**) hepatocellular carcinoma occurrence (**b**) HBsAg seroclearance, and (**c**) overall mortality in the coexistence group and the control group.

HCC was diagnosed in 37 patients (1.7%) in the control group and 6 patients (3.6%) in the coexistence group during the follow-up period. Based on the Kaplan-Meier analysis, a marginal association was observed between the coexistence of HBsAg and anti-HBs and HCC development (log-rank test, *P* = 0.071). In the multivariate Cox regression analysis, the coexistence of HBsAg and anti-HBs resulted in a 3.1-fold higher risk of HCC occurrence than the presence of HBsAg alone (hazard ratio (HR) 3.08, 95% confidence interval (CI) 1.26–7.55, *P* = 0.014). Male sex (HR 4.92, 95% CI 1.74–13.89, *P* = 0.003), presence of cirrhosis (HR 7.30, 95% CI 3.50–15.24, *P* < 0.001), significant fibrosis evaluated by FIB-4 (HR 4.52, 95% CI 2.10–9.74, *P* < 0.001), higher qHBsAg (HR 2.80, 95% CI 1.10–7.15, *P* = 0.032) were also significantly associated with HCC occurrence (Table 2 and Fig. 2a).

**Table 2:** Univariate and multivariate analyses by Cox proportional hazard regression for hepatocellular carcinoma occurrence.

	Univariate analysis		Multivariate analysis	
	HR (95% CI)	*P*-value	HR (95% CI)	*P*-value
Age >50 years	2.28 (1.25–4.15)	0.007	1.24 (0.62–2.48)	0.539
Male sex	6.00 (2.14–16.78)	0.001	4.92 (1.74–13.89)	0.003
Coexistence of HBsAg and anti-HBs	2.22 (0.93–5.27)	0.071	3.08 (1.26–7.55)	0.014
ALT	1.004 (1.003–1.006)	<0.001	1.00 (1.00–1.01)	0.146
Presence of cirrhosis	15.46 (8.14–29.36)	<0.001	7.30 (3.50–15.24)	<0.001
FIB-4 > 1.45	7.45 (3.82–14.50)	<0.001	4.52 (2.10–9.74)	<0.001
Treatment with antiviral agent	2.73 (1.37–5.41)	0.004	1.05 (0.49–2.24)	0.895
qHBsAg > 250 IU/ml	1.60 (0.67–3.81)	0.293	2.80 (1.10–7.15)	0.032

ALT, alanine aminotransferase; FIB-4, fibrosis-4; qHBsAg, quantitative HBsAg; HR, hazard ratio; CI, confidence interval.

In the PS matched cohort, most variables were balanced between groups (Supplementary Table 1). The PS matched analysis also showed that coexistence of HBsAg and anti-HBs were independent risk factor for HCC development (HR 10.20, 95% CI 1.10–92.00, log-rank test = 0.015).

### HBsAg seroclearance

HBsAg seroclearance was observed in 316 patients: 39/166 (23.5%) in the coexistence group, and 277/2175 (12.7%) in the control group. The cumulative probabilities of HBsAg seroclearance in the total population were 1.24% at 1 year, 5.75% at 3 years, and 10.78% at 5 years. The annual HBsAg seroclearance rate was 4.92% in the coexistence group and 2.56% in the control group.

Based on the Kaplan-Meier analysis, the coexistence group was associated with more HBsAg seroclearance (log-rank test < 0.001). The association between the coexistence of HBsAg and anti-HBs and HBsAg seroclearance (HR 1.43, 95% CI 1.01–2.03, *P* = 0.046) remained significant even after adjustment for covariates including age, sex, ALT level, presence of cirrhosis, significant fibrosis evaluated by FIB-4, history of antiviral medication, and qHBsAg level (Table 3 and Fig. 2b).

**Table 3:** Univariate and multivariate analyses by Cox proportional hazard regression for HBsAg seroclearance.

	Univariate analysis		Multivariate analysis	
	HR (95% CI)	*P*-value	HR (95% CI)	*P*-value
Age > 50 years	1.61 (1.29–2.02)	<0.001	0.83 (0.64–1.07)	0.148
Male sex	1.63 (1.28–2.08)	<0.001	1.28 (0.99–1.66)	0.060
Coexistence of HBsAg and anti-HBs	1.93 (1.38–2.70)	<0.001	1.43 (1.01–2.03)	0.046
ALT	1.00 (0.99–1.00)	0.064	1.00 (0.99–1.00)	0.132
Presence of cirrhosis	1.26 (0.71–2.25)	0.432	1.26 (0.69–2.28)	0.453
FIB-4 > 1.45	1.21 (0.95–1.54)	0.132	1.20 (0.92–1.57)	0.185
Treatment with antiviral agent	0.26 (0.14–0.47)	<0.001	0.41 (0.22–0.79)	0.007
qHBsAg > 250 IU/ml	0.06 (0.05–0.07)	<0.001	0.06 (0.05–0.08)	<0.001

ALT, alanine aminotransferase; FIB-4, fibrosis-4; qHBsAg, quantitative HBsAg; HR, hazard ratio; CI, confidence interval.

### All-cause mortality

As shown in Table 4 and Fig. 2c, no significant difference was observed in overall mortality between the coexistence group and the control group. Older age (*P* = 0.010) and the presence of cirrhosis (*P* = 0.002) were predictive factors for overall mortality.

**Table 4:** Univariate and multivariate analyses by Cox proportional hazard regression for overall mortality.

	Univariate analysis		Multivariate analysis	
	HR (95% CI)	*P*-value	HR (95% CI)	*P*-value
Age >50 years	3.26 (1.65–6.45)	0.001	2.75 (1.28–5.90)	0.010
Male sex	1.72 (0.80–3.69)	0.163	1.62 (0.75–3.54)	0.222
Coexistence of HBsAg and anti-HBs	1.25 (0.38–4.10)	0.710	1.54 (0.46–5.13)	0.481
ALT	1.00 (1.00–1.01)	0.489	1.00 (1.00–1.01)	0.731
Presence of cirrhosis	5.37 (2.08–13.87)	0.001	4.88 (1.75–13.61)	0.002
FIB-4 > 1.45	2.76 (1.41–5.40)	0.003	1.67 (0.77–3.62)	0.197
Treatment with antiviral agent	0.30 (0.04–2.19)	0.235	0.16 (0.02–1.20)	0.075
qHBsAg > 250 IU/ml	1.11 (0.46–2.67)	0.823	1.67 (0.67–4.16)	0.268

ALT, alanine aminotransferase; FIB-4, fibrosis-4; qHBsAg, quantitative HBsAg; HR, hazard ratio; CI, confidence interval.

## Discussion

This study evaluated the long-term clinical outcomes of the coexistence of HBsAg and anti-HBs. The proportion of HBsAg/anti-HBs double-positive chronic hepatitis B patients was 7.0% among in this study. The coexistence of HBsAg and anti-HBs was associated with a higher HCC risk and more HBsAg seroclearance than the presence of HBsAg alone. Nevertheless, overall mortality was not significantly different between the coexistence and control groups.

Our study showed a higher HBsAg/anti-HBs double positivity rate than that reported by previous studies (2.8% to 3.6%)^2–5^. This might be due to the detection of double positivity during the follow-up duration. A previous longitudinal study similarly reported a HBsAg/anti-HBs double positivity rate of 7.1%^13^. Differences in the characteristics of study populations can also affect the prevalence of HBsAg and anti-HBs double positivity. For example, pre-S deletion, which is thought to be associated with the coexistence of HBsAg and anti-HBs, has been found more frequently in chronic hepatitis B patients with genotype C than in those with genotype B^14,15^.

There are several plausible explanations for the coexistence of HBsAg and anti-HBs. First, selection of immune escape mutants might occur during chronic HBV infection despite the production of anti-HBs^1^. Mutations or deletions in the pre-S/S gene^16^, mutation in “a” determinant and in other regions of the “S” protein^17^ or emergence of heterologous subtype-specific antibodies directed against HBsAg subtypes different from coexisting HBsAg^8,18,19^ can explain this phenomenon^1^. Second, superinfection with a new HBV strain is another possible explanation^1,20^. Infection with an HBV escape mutant has also been reported in the presence of anti-HBs even after vaccination^20,21^. Third, HBV reactivation during occult HBV infection often associated with immunosuppressive conditions is another possible mechanism^22,23^. Fourth, false positivity for anti-HBs antibody could explain the coexistence of HBsAg and anti-HBs, and this phenomenon can occur as a result of exposure to diverse naturally developing antigens or to other infections, such as *Escherichia coli* or *Neiserria gonorrhoeae*^24^. Sample storage or specimen handling might also affect the false positivity of anti-HBs. Fifth, the coexistence of HBsAg and anti-HBs may represent a possible transition from HBsAg to recovery to anti-HBs. In this case, the level of HBsAg and anti-HBs is usually very low, and the HBsAg/anti-HBs double-positive status might be transient. Previous studies have also reported HBsAg seroreversion (reappearance of HBsAg after HBsAg seroclearance) in patients who experienced HBsAg seroclearance with positive anti-HBs^25,26^. Ozeki *et al*. reported 2 cases with temporarily or continuously positive anti-HBs during HBsAg seroreversion, and HBsAg seroclearance was again observed in these cases^27^. Wong *et al*. reported 1 patient among 6 patients with seroreversion who developed anti-HBs^28^. This patient had undetectable HBV DNA even after seroreversion to a HBsAg-positive state. By evaluated HBsAg seroclearance after nucleoside analog therapy, another study showed that HBsAg seroreversion occurred in some patients who experienced HBsAg seroclearance, most of which were transient with extremely low serum levels of HBsAg and HBV DNA^25^. According to these different mechanisms, the impact of HBsAg/anti-HBs double positivity on outcomes could be different. However, the evaluation of viral mutations and genetic subtypes was not performed in this study. Thus, we could not determine the proportion of subjects affected by each of these possible underlying mechanisms or determine the main underlying mechanism for the coexistence of HBsAg and anti-HBs.

According to our results, the coexistence of HBsAg and anti-HBs was associated with HCC development. This result is consistent with the findings of a previous smaller study with a median of 4.3 years follow-up, which reported a 2-fold higher HCC occurrence rate in the coexistence group than HBsAg-only positive group^13^. Our study with 2,341 patients with an approximately 5 year follow-up showed similar results, reporting a 3.1-fold higher risk of HCC in the coexistence group than in the control group independent of other risk factors for HCC. As deletion of pre-S1 regions, one of possible mechanism for the HBsAg and anti-HBs double positive, has been suggested as a risk factor in progressive liver disease, including hepatocarcinogenesis^15,29,30^, this might explain the increase in HCC occurrence in the coexistence group. Further study evaluating the mutational status of pre-S1 in the coexistence and control groups might help to evaluate whether this phenomenon can explain the increased HCC incidence in the coexistence group.

Intriguingly, the coexistence of HBsAg and anti-HBs was also associated with increased HBsAg seroclearance, which is associated with good prognosis^25,31,32^. One plausible explanation for more HBsAg seroclearance is that patients who had anti-HBs after seroconversion and then experienced transient low-level HBsAg seroreversion were included in the coexistence group as presented above. As these patients usually have very low levels of HBsAg and tend to experience repeated seroclearance of HBsAg^25,27^, this might explain better HBsAg seroclearance in the coexistence group than in the control group. Another plausible explanation is that seroclearance of HBsAg is not indicative viral clearance but rather of a point mutation in the S gene that results in the failure to detect HBsAg^33,34^. In this study, only 10% of subjects (4 out of 39 patients) with HBsAg seroclearance in the coexistence group were subjected to HBV DNA level analysis at the time of seroclearance, and these 4 patients showed no or minimal level of HBV DNA (less than 20 IU/mL), suggesting true HBsAg seroclearance with viral clearance rather than detection failure.

The coexistence of HBsAg and anti-HBs was associated with a risk of HCC occurrence, which represents poor outcome. However, the coexistence of HBsAg and anti-HBs was also associated with HBsAg seroclearance, which represents a favorable outcome. Additionally, these events were mutually exclusive. In other words, in the coexistence group, 6 patients developed HCC, and 39 patients experienced HBsAg seroclearance during follow-up. However, no patient experienced both HBsAg seroclearance and HCC occurrence. This result suggests that the coexistence group is heterogeneous. More detailed serological analyses, including viral mutations, might help identify differences in these populations.

This study has several strengths. First, this study is the first to evaluate the long-term outcomes of HBsAg/anti-HBs double-positive patients from several aspects, including HCC occurrence, HBsAg seroclearance, and overall mortality. Second, HBsAg was evaluated nearly annually in most patients with a mean of 4.4 times per patient over a 5-year follow-up period. As the level of HBsAg and anti-HBs is variable, the change in HBsAg and anti-HBs levels could be evaluated with this regular repeated follow-up.

This study has several limitations. First, although this study described longitudinal outcomes of patients positive for both HBsAg and anti-HBs, the underlying mechanism was not determined in this study, as mentioned above. Further studies, such as the evaluation of pre-S1 mutations, are warranted to evaluate the mechanism underlying higher HCC incidence or more HBsAg seroclearance in the coexistence group than in the control group. Second, a sufficient serological profile reflecting HBV infection status, such as HBeAg, anti-HBe and HBV DNA, was not evaluated in all subjects because of the nature of our health screening policy. Third, only Korean patients of a single ethnic origin, more than 99% of whom were infected by genotype C HBV, were included in this study^35^. To generalize these findings, validation in other ethnic groups with variable genotypes might be necessary.

In conclusion, this study shows the long-term outcomes of patients positive for both HBsAg and anti-HBs. The coexistence of HBsAg and anti-HBs was associated with higher HCC incidence rates and more HBsAg seroclearance than the presence of HBsAg alone. Nevertheless, overall mortality was not significantly different between the coexistence group and the control group. It suggests that the coexistence group could not be defined as one homogenous group, but consists of groups with favorable outcomes and groups with unfavorable outcomes.

### Ethical approval and informed consent

This study protocol conformed to the ethical guidelines of the 1975 Declaration of Helsinki and was approved by the Institutional Review Board of Seoul National University Hospital (H-1704-078-846). The requirement for informed consent from subjects was waived because the researchers accessed only deidentified database entries for analytical purposes.

### Grant support

This study was supported by grant 04-2017-0730 (2017–1131) from the Seoul National University Hospital Research Fund. The funding organizations played no role in the design or implementation of the study, the collection, analysis, or interpretation of the data, or the writing, review, or approval of the manuscript.

## Supplementary information

Supplementary file
