# Supplementary material for: Long-term outcomes of HBsAg/anti-HBs double-positive versus HBsAg single-positive patients with chronic hepatitis B
Source: Sci Rep. 2019 Dec 19;9:19417. doi: 10.1038/s41598-019-56015-8 (PMC6923451; doi:10.1038/s41598-019-56015-8)

Supplementary Files

Long-term outcomes of HBsAg/anti-HBs double-positive versus HBsAg single-positive patients with chronic hepatitis B

Min-Sun Kwak*, MD. PhD.[1], Goh-Eun Chung, MD. PhD.[1], Jong In Yang, MD. PhD.[1], Jeong Yoon Yim, MD. PhD.[1]

Supplementary Table 1. Baseline characteristics of subjects with coexistence of HBsAg and anti-HBs compared to subjects with positive HBsAg in propensity score-matched cohort

	Control group	Coexistence group	*P*-value
n	110	110	
Age, years	47.5 ± 10.0	46.0 ± 9.2	0.244
Male sex	69 (62.7%)	70 (36.4%)	0.889
Body mass index, m/kg^2	23.8 ± 3.3	23.7 ± 2.9	0.834
Alcohol consumption	84 (76.4%)	86 (78.2%)	0.748
Smoking			0.192
Never	56 (50.9%)	46 (41.8%)	
Former	26 (23.6%)	38 (34.6%)	
Current	28 (25.5%)	26 (23.6%)	
Diabetes	12 (10.9%)	7 (6.4%)	0.230

qHBsAg > 250 IU/ml	70 (63.6%)	74 (67.3%)	0.571
WBC, /µL	5,557 ± 1,535	5,483 ± 1,601	0.726
Hemoglobin, g/dL	14.6 ± 1.5	14.6 ± 1.8	0.911
Platelet, x10^3/µL	211.7 ± 52.0	216.5 ± 54.8	0.506
Cholesterol	181.1 ± 35.0	183.1 ± 31.3	0.661
Total protein	7.2 ± 0.4	7.1 ± 0.4	0.217
Albumin	4.3 ± 0.3	4.3 ± 0.3	0.962
Bilirubin	1.1 ± 0.4	1.1 ± 0.4	0.244
Alkaline phosphatase	62.9 ± 18.6	61.6 ± 16.7	0.576
AST	35.4 ± 42.3	34.4 ± 35.6	0.855
ALT	41.4 ± 61.3	40.9 ± 50.2	0.952
GGT	38.7 ± 42.4	35.5 ± 29.5	0.509
FIB-4 > 1.45	30 (27.3%)	26 (23.6%)	0.536
Presence of cirrhosis	1 (0.9%)	2 (1.8%)	0.561
Treatment with anti-viral agent	11 (10.0%)	12 (10.9%)	0.826

WBC, white blood cell; AST, aspartate aminotransferase; ALT, alanine aminotransferase; GGT, gamma glutamyl transpeptidase; FIB-4, fibrosis-4

Supplementary Figure 1. The dynamic change of HBsAg and anti-HBs of the patients with coexistence of HBsAg and anti-HBs. The horizontal axis represents the number of serologic tests including HBsAg and anti-HBs. The vertical axis represents each patient. The red color indicates double positive HBsAg and anti-HBs. The orange color indicates HBsAg only positive, the blue color indicates negative HBsAg and positive anti-HBs, and the green color indicates negative HBsAg and negative anti-HBs.

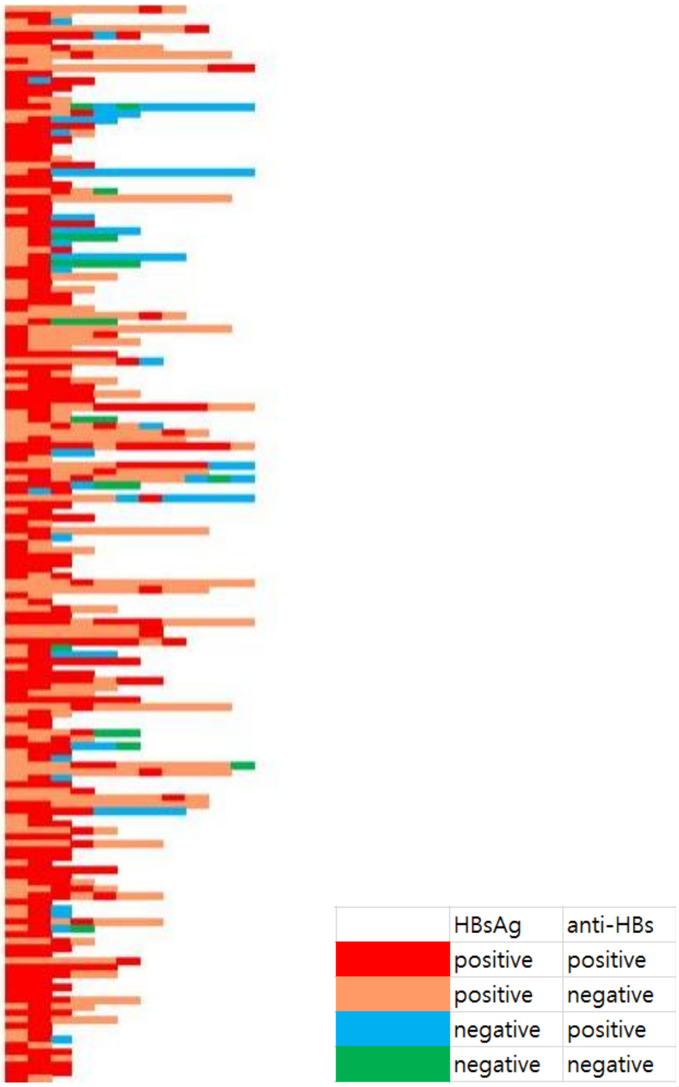

Supplement: Supplementary file 1 — Supplementary file [file 41598_2019_56015_MOESM1_ESM.pdf]
